# Quality of Life, Depression, Anxiety Symptoms and Mood State of Wheelchair Athletes and Non-athletes: A Preliminary Study

**DOI:** 10.3389/fpsyg.2019.01848

**Published:** 2019-08-13

**Authors:** Rodrigo Luiz Vancini, Andressa Amato Gomes, Hudson de Paula-Oliveira, Claudio de Lira, Weverton Rufo-Tavares, Marilia Santos Andrade, Karine Jacon Sarro, Martoni Moreira Sampaio, Ricardo Borges Viana, Pantelis Theodoros Nikolaidis, Thomas Rosemann, Beat Knechtle

**Affiliations:** ^1^Centro de Educação Física e Desportos, Laboratório de Força e Condicionamento, Universidade Federal do Espírito Santo, Vitória, Brazil; ^2^Setor de Fisiologia Humana e do Exercício, Laboratório de Avaliação do Movimento Humano, Faculdade de Educação Física e Dança, Universidade Federal de Goiás, Goiânia, Brazil; ^3^Departamento de Fisiologia, Universidade Federal de São Paulo, São Paulo, Brazil; ^4^Faculdade de Educação Física, Universidade Estadual de Campinas, Campinas, Brazil; ^5^Secretaria de Estado de Esportes e Lazer (SESPORT), Vitória, Brazil; ^6^Exercise Physiology Laboratory, Nikaia, Greece; ^7^Institute of Primary Care, University of Zurich, Zurich, Switzerland; ^8^Medbase St. Gallen Am Vadianplatz, St. Gallen, Switzerland

**Keywords:** quality of life, depression, anxiety, mood state, wheelchair, athletes

## Abstract

The present study aims to compare quality of life, depression, anxiety symptoms, and profile of mood state of wheelchair athletes and non-athletes. Thirty-nine basketball and rugby wheelchair athletes (*n* = 23, nine women, age 36.0 ± 10.0 years; body mass 66.2 ± 13.8 kg; height 170.0 ± 8.5 cm) and non-athletes (*n* = 16, 4 women, 39.0 ± 14.2 years; body mass 79.6 ± 17.2 kg; height 170.0 ± 6.4 cm) were recruited. Quality of life, anxiety and depressive symptoms and mood disorders were evaluated by the Medical Outcomes Short-Form Health Survey (SF-36), State-Trait Anxiety Inventory, Beck Depression Inventory and Profile of Mood State questionnaire, respectively. Comparison between groups (non-athletes vs. athletes) was performed using Student’s *t*-test for independent samples. No differences (*p* > 0.05) were found between non-athletes vs. athletes regards to quality of life, depressive and anxiety symptoms and profile of mood state. Overall, non-athletes and athletes presented medium anxiety symptoms and mild to moderate depressive symptoms. In conclusion, the wheelchair athletes and non-athletes presented similar quality of life, depressive and anxiety symptoms, and profile of mood state.

## Introduction

There is a growing number of people with disabilities (including wheelchair users) who participate in regular physical activity/sports programs with ludic and rehabilitation purposes as well as with elite sport performance purposes (Bhambhani, 2002; De Lira et al., 2010; Lee and Uihlein, 2019). Indeed, the participation of people with disabilities in sports is desirable, because it has a positive impact on social, psychological and physical aspects (Côté-Leclerc et al., 2017). Moreover, it is considered as a complementary strategy for physical, social and emotional rehabilitation, and consequently, could improve the quality of life (Blauwet and Willick, 2012; Lee and Uihlein, 2019). Furthermore, wheelchair sports consist a favorable opportunity for people with disabilities by increasing the possibility of social integration and physical, motor, psychological, and neurological rehabilitation (Lee and Uihlein, 2019).

Despite of indisputable benefits of exercise and sports for people with disabilities, there are some barriers that prevent the participation of people with disabilities (including wheelchair users) in adapted sports. These barriers could be divided into psychological, physical, physiologic, and environmental factors (Côté-Leclerc et al., 2017). For instance, Côté-Leclerc et al. (2017) pointed that physical and mobility limitations might restrict opportunities to perform sports and physical activity, which may affect quality of life. Regarding to psychological factors, in general, people with disabilities have low self-esteem and confidence, decreased motivation, increased depression and pain, and high stress level which affect negatively quality of life (Lee and Uihlein, 2019).

Previous meta-analysis showed that exercise can improve mental health (Ochentel et al., 2018; Rodriguez-Ayllon et al., 2019) and quality of life (Conn et al., 2009; Gillison et al., 2009; Sweegers et al., 2018) in healthy and clinical population. For example, Schuch et al. (2018) showed that exercise can confer protection against the emergence of depression regardless of age and geographical region. Conn et al. (2009) conducted a meta-analysis to investigate the effects of interventions to increase physical activity among adults with chronic illness in quality of life outcomes and found that, despite considerable heterogeneity in the magnitude of the effect, participants improved quality of life from exposure to interventions designed to increase physical activity. However, there are, so far as we are aware, few studies that investigated whether participation in adapted sports (particularly wheelchairs sports) affects the quality of life and emotional status of people with disabilities. For example, Muraki et al. (2000) demonstrated that sports activity can improve the psychological status in both tetraplegics and paraplegics with spinal cord injury, and the psychological benefits are emphasized by sports activity at high frequency.

Considering that about 10% of worldwide population (∼650 million people) have disabilities and that about 10% of people with disabilities require a wheelchair, studies that investigated the effects of exercise on mental health and quality of life of wheelchair are warranted (World Health Organization, 2011). Thus, the aim of the present study was to compare the quality of life, depressive and anxiety symptoms, and profile of mood state between wheelchair users practicing sports or not. The research hypothesis is that people with disabilities, especially those wheelchair users that not practicing sports, would present poor levels of quality of life, anxiety and depressive symptoms, a negative profile of mood state as compared with wheelchair users practicing sports.

## Materials and Methods

### Participants

Basketball and rugby wheelchair athletes and non-athletes participated in this study. Due to difficulties in recruiting and assessing wheelchair users (sports and/or non-sports practitioners), the sample consisted of people of both sexes with different types of affections (the most commons were spinal cord injury and neurological diseases).

Initially, 50 wheelchair users (25 athletes and 25 non-athletes) were invited to participate. However, two athletes and nine non-athletes did not accept or gave up participation in the study. Thus, the final sample was constituted by 39 participants (26 men and 13 women), 23 in the athlete group (14 men and nine women) and 16 in the non-athlete group (12 men and four women). The athlete group were recruited from the teams (basketball and rugby wheelchair) of the *Physical Rehabilitation Center of Espírito Santo (Espírito Santo, Brazil)*. Regarding to physical training characteristics of athletes group, 11 (47.8%) trained 3 h and 3 days/week, 2 (8.7%) trained 3 h and 6 days/week, 6 (26.1%) trained 4 h and 3 days/week, 3 (13%) trained 5 h and 3 days/week, and 1 (4.3%) trained 6 h and 3 days/week. Relative to participation in competitions, 19 (82.6%) had participated and 4 (17.4%) had not participated. Participants in the non-athlete group did not practice sports.

The participants of the non-athlete group were recruited from several places, such as therapeutic centers, rehabilitation clinics, universities and private companies. Age, body mass and height were self-reported by the participants at the interview time application (Table 1). Concerning the athletes, 23 people were interviewed being: 9 (39.1%) practitioners of wheelchair rugby and 14 of wheelchair basketball (60.9%); 9 were women (39.1%) and 14 (60.9%) were men; 12 (52.2%) were unmarried, 6 (26.1%) married, 3 (13%) divorced and 2 (8.7%) maintained a stable union; 5 (21.7%) had work and 18 (78.3%) were unemployed/retired; and 4 (17.4%) had incomplete elementary education, 3 (13%) had only completed elementary education, 1 (4.3%) had incomplete secondary education, 12 (53.2%) had high school and 3 (13%) were either attending or had completed higher education.

**TABLE 1:** General characteristics of the non-athlete and athlete groups.

	**Non-athlete group (*n* = 16)**			**Athlete group (*n* = 23)**			***p*-value**
			
	**Mean ± SD**	**Median [q1-q3]**	**Min-Max**	**Mean ± SD**	**Median [q1-q3]**	**Min-Max**	
Age (years)	39.0 ± 14.2	33 [30–51]	20–67	36.0 ± 10.0	35 [30–38]	19–57	0.461
Body mass (kg)	79.6 ± 17.2	76 [65–90]	53–115	66.2 ± 13.8^*^	66 [56–72]	38–106	0.014
Height (cm)	170.0 ± 6.4	170 [165–175]	162–182	170.0 ± 8.5	170 [165–174]	148–181	0.564

SD, standard deviation. ^*^Significant different from non-athlete group.

For the non-athletes, 16 people were interviewed, being: 12 (75%) males and 4 (25%) females; 11 (68.8%) were unmarried, 4 (25%) were married and 1 (6.3%) were divorced; 4 (25%) worked and 12 (75%) were unemployed/retired; and 1 (6.3%) had incomplete elementary education, 2 (12.5%) had completed elementary education, 6 (37.5%) had completed high school, 4 (25.5%) had incomplete 1 (6.3%) had completed higher education. Both groups (non-athletes and athletes) presented similar socio-demographic characteristics (Table 2).

**TABLE 2:** Socio-demographic characteristics of non-athlete and athlete groups.

	**Non-athlete group (*n* = 16)**	**Athlete group (*n* = 23)**	**Statistics**	***p*-value**
**Sex**				
Men (*n* = 26)	12(46.2%)	14(53.8%)	X^2^(1) = 0.848	0.357
Women (*n* = 13)	4(30.8%)	9(69.2%)		
**Marital status**				
Single (*n* = 23)	11(47.8%)	12(52.2%)		0.520
Married (*n* = 10)	4(40%)	6(60%)	X^2^(3) = 2.260	
Divorced (*n* = 4)	1(25%)	3(75%)		
Stable union (*n* = 2)	0(0%)	2(100%)		
**Work**				
Yes (*n* = 9)	4(44.4%)	5(55.6%)	X^2^(1) = 0.057	0.812
No (*n* = 30)	12(40%)	18(60%)		
**Education level**				
Elementary school (*n* = 11)	3(27.3%)	8(72.7%)		0.538
High school (*n* = 22)	10(45.5%)	12(54.5%)	X^2^(2) = 1.238	
Higher education (*n* = 6)	3(50%)	3(50%)		

All participants were informed about the aims, procedures, possible discomforts, risks and benefits of the study, and signed the informed consent form. All the experimental procedures were submitted and approved by the Ethics Committee of the Federal University of Espírito Santo (CAAE: 70119817.3.0000.5542 and protocol number: 2.182.183) and are in accordance with the Declaration of Helsinki.

### Research Procedures

This was a cross-sectional study conducted in 1 day. Both groups (athletes and non-athletes groups) answered the following questionnaires in this order: (i) a questionnaire for collecting demographic and exercise characteristics data; (ii) Medical Outcomes Study 36-Item Short-Form Health Survey (SF-36); (iii) State-Trait Anxiety Inventory (STAI); (iv) Beck Depression Inventory (BDI); and (v) Profile of Mood State questionnaire (POMS) in order to evaluate demographic and exercise characteristics (weekly frequency, duration and workload), health-related quality of life, anxiety symptoms, depressive symptoms, and profile of mood state, respectively.

### Quality of Life Assessment

The perception of quality of life was evaluated using the SF-36, translated and validated for Brazilian Portuguese by Ciconelli et al. (1999). The SF-36 is a widely used, generic questionnaire that comprises eight subscales: functioning capacity (10 items), role limitations due to physical problems (four items), pain (two items), general health perceptions (five items), vitality (four items), social functioning (two items), role limitations due to emotional problems (three items), and mental health (five items). In addition, a single item provides an indication of perceived change in health (Ware and Sherbourne, 1992). Subscale scores range from 0 to 100, with 100 being the best, most positive, quality of life on the subscale measured and 0 the worst. The questionnaire was conducted as an interview. Internal consistency of the SF-36 is good, with Cronbach’s alpha ranging from 0.76 to 0.90 for all subscales of the questionnaire (Jenkinson et al., 1994). Froehlich-Grobe et al. (2008) showed that the SF-36 successfully measures (good fit) health-related quality of life among mobility-impaired individuals, including wheelchair users.

### Assessment of Anxiety and Depressive Symptoms

To assess the anxiety symptoms, the STAI, translated and adapted into Brazilian Portuguese by Biaggio et al. (1977), was used. Briefly, the questionnaire consists of two different scales of anxiety, one that evaluates state and another that evaluates trait. The trait-scale consists of 20 statements related to how the participant usually feels, and state-scale consists of 20 statements regarding the current situation of the participant. Both scales classify the participant as having low, medium or high levels of anxiety. Scores can vary from of 20 to 80. A score ≤ 30 indicates a low level of anxiety, a score between 31 and 49 a medium level, and a score equal to or greater than 50 a high level of anxiety. Internal consistency coefficients for the scale range from 0.86 to 0.95; and test-retest reliability coefficients range from 0.65 to 0.75 over a 2-month interval (Spielberger et al., 1983).

To assess the depressive symptoms, the BDI, translated and adapted to Brazilian Portuguese by Gorenstein and Andrade (1996), was used. BDI is one of the most frequently used questionnaires to evaluate the severity of depressive symptoms. This instrument has 21 questions about symptoms of depression that cover affective, behavioral, somatic and interpersonal aspects. Each item consists of a series of four statements scaled to indicate increasing depressive symptomatology. The classification of the score ranges from normal to mild, mild to moderate and moderate to severe depressive symptoms. Scores below 9 are considered normal, whereas scores between 10 and 18 indicate mild to moderate depressive symptoms, 19 to 29 indicate moderate to severe depressive symptoms, and 30 to 63 severe depressive symptoms. Internal consistency of BDI ranges from 0.81 (for students) to 0.88 (for depressed patients) (Beck et al., 1979; Gorenstein and Andrade, 1996).

### Profile of Mood State Evaluation

Mood state was assessed by the POMS (Morgan et al., 1987, 1988; Peluso and Guerra de Andrade, 2005), which is a self-report, global mood measure comprising 65 items across six categories: anxiety-tension, depression, anger-hostility, vigor, fatigue, and confusion and scored from 1 to 4 according to severity. The questionnaire yields a global measure of mood. The global score is computed by subtracting the positive category (vigor) from the sum of the five negative categories (tension, depression, anger, fatigue, and confusion). The number 100 was added to the final TMD score to avoid negative results (Legey et al., 2016).

### Statistical Analysis

Data was presented as mean, median, interquartile range, standard deviation, minimum, and maximum. The normality was evaluated by the Shapiro–Wilk test. As the data presented normal distribution, comparison between groups (non-athletes vs. athletes) was performed using Student’s *t*-test for independent samples. Chi-square tests were performed on categorical variables (socio-demographic characteristics and signs and symptoms of depression and anxiety) to determine differences in proportion between the non-athletes vs. athletes. The level of significance adopted in all analyzes was 5% with the 95% confidence interval. The Statistical Package for the Social Sciences (SPSS) version 21.0 (IBM Corp., Armonk, NY, United States) was used for all statistical analysis.

## Results

Regarding to the evaluation of quality of life, depressive and anxiety symptoms and profile of mood state, no significant differences were found between the mean scores of the non-athlete and athlete groups (Tables 3, 4). Nevertheless, the values of the domains of quality of life evaluated, in general, were numerically higher for the athlete group. However, this pattern was not repeated for depressive and anxiety symptoms and profile of mood state.

**TABLE 3:** Quality of life profile (SF-36) of non-athlete and athlete groups.

**SF-36 domains**	**Non-athlete group (*n* = 16)**			**Athlete group (*n* = 23)**			***p*-value**
			
	**Mean ± SD**	**Median [q1-q3]**	**min-max**	**Mean ± SD**	**Median [q1-q3]**	**min-max**	
Functional capacity	44.4 ± 28.1	47 [13–71]	0–80	56.3 ± 23.6	55 [45–70]	5–100	0.160
Limitations by physical aspects	54.6 ± 34.4	62 [25–75]	0–100	65.2 ± 39.7	75 [25–100]	0–100	0.396
Pain	71.1 ± 25.2	73 [51–96]	22–100	72.0 ± 19.5	72 [51–80]	51–80	0.591
General health status	73.1 ± 22.9	77 [52–95]	27–100	77.0 ± 22.7	87 [62–97]	20–100	0.607
Vitality	68.4 ± 18.3	75 [55–80]	35–100	69.3 ± 18.6	75 [65–80]	20–95	0.881
Social aspects	72.6 ± 22.9	62 [50–100]	37–100	80.0 ± 25.2	87 [75–100]	12–100	0.332
Limitations due to emotional problems	66.7 ± 34.4	66 [50–100]	0–100	73.9 ± 46.2	100 [33–100]	0–100	0.537
Mental health	79.5 ± 12.3	78 [72–91]	60–100	79.4 ± 16.0	84 [76–88]	20–100	0.996

*SF-36: short form 36 questionnaire. SD, standard deviation.*

**TABLE 4:** Profile of mood state (POMS) and depressive and anxiety symptoms of non-athlete and athlete groups.

	**Non-athlete group (*n* = 16)**			**Athlete group (*n* = 23)**			***p*-value**
			
	**Mean ± SD**	**Median [q1-q3]**	**min-max**	**Mean ± SD**	**Median [q1-q3]**	**min-max**	
POMS							
Tension-anxiety	4.8 ± 4.5	5[2−6]	−2–15	4.0 ± 6.6	2[0−5]	−3–26	0.675
Depression	2.6 ± 2.9	2[0.25−4]	0–9	5.3 ± 10.5	1[0−4]	0–45	0.332
Anger-hostility	6.5 ± 4.3	6[3−11]	0–14	7.4 ± 9.8	4[1−11]	0–44	0.712
Vigor	18.7 ± 5.0	20[15−21]	0–10	19.2 ± 5.3	20[18−24]	7–29	0.786
Fatigue	4.1 ± 2.7	4[2−5]	0–10	4.7 ± 5.4	3[1−7]	0–22	0.660
Confusion	1.1 ± 2.6	1[0.75−2]	−3–6	2.0 ± 5.0	0[-*1*-5]	−4–17	0.528
Disturbance mood total score	100.5 ± 13.2	102[94−111]	73–118	104.4 ± 38.3	92[80−110]	74–247	0.656
Anxiety-Trait	40.0 ± 7.3	41[35−45]	25–51	37.8 ± 10.3	36[31−43]	23–71	0.476
Anxiety-State	36.6 ± 7.8	35[30−41]	24–57	37.5 ± 6.8	35[33−39]	31–60	0.706
Depressive symptoms	12.8 ± 6.4	11[7−18]	4–23	13.3 ± 7.3	13[9−16]	3–36	0.821

*SD, standard deviation.*

Regarding to non-athlete group, 37.5% (*n* = 6), 25.0% (*n* = 4), 18.8% (*n* = 3), and 18.8% (*n* = 3) presented normal, slight, slight to moderate, and moderate to severe depressive symptoms, respectively. For anxiety-Trait and State of the non-athlete group, 12.5% (*n* = 2) and 25.0% (*n* = 4), 81.3% (*n* = 13) and 68.8% (*n* = 11), and 6.3% (*n* = 1) and 6.3% (*n* = 1) presented low, mild and high levels, respectively. Relative to athlete group, 39.1% (*n* = 9), 30.4% (*n* = 7), 21.7% (*n* = 5), and 8.7% (*n* = 2) presented normal, slight, slight to moderate and moderate to severe depressive symptoms, respectively. Concerning to anxiety-trait of the athlete group, 21.7% (*n* = 5), 65.2% (*n* = 15), and 13.0% (*n* = 3) presented low, medium and high levels, respectively. For anxiety-State of the athlete group, 95.7% (*n* = 22) and 4.3% (*n* = 1) presented mild and high levels, respectively.

Chi-square test revealed a significant difference in the proportion between the non-athletes vs. athletes with low, medium and high anxiety-State (Table 5); however, no significant differences were found between non-athletes and athletes in anxiety-Trait, depressive symptoms (Table 5) or socio-demographic characteristics (Table 2).

**TABLE 5:** Classification of signs and symptoms of depression and anxiety of non-athlete and athlete groups.

	**Non-athlete group (*n* = 16)**	**Athlete group (*n* = 23)**	**Statistics**	***p*-value**
**Depressive symptoms**				
Normal (*n* = 15)	6 (40.0%)	9 (60.0%)		
Slight (*n* = 11)	4 (36.4%)	7 (63.6%)	X^2^(3) = 0.890	0.828
Slight to moderate (*n* = 8)	3 (37.5%)	5 (62.5%)		
Moderate to severe (*n* = 5)	3 (60.0%)	2 (40.0%)		
**Anxiety-Trait**				
Low (*n* = 7)	2 (28.6%)	5 (71.4%)		
Medium (*n* = 28)	13 (46,4%)	15 (53.6%)	X^2^(2) = 1.211	0.546
High (*n* = 4)	1 (25.0%)	3 (75.0%)		
**Anxiety-State**				
Low (*n* = 4)	4 (100%)	0 (0%)		
Medium (*n* = 33)	11 (33.3%)	22 (66.7%)	X^2^(2) = 6.624	0.036
High (*n* = 2)	1 (50.0%)	1 (50.0%)		

## Discussion

The aim of the present study was to evaluate the quality of life, depression and anxiety symptoms and profile of mood state of wheelchair users that practicing sports or not. The research hypothesis is that people with disabilities, especially those wheelchair users that not practicing sports, would present poor levels of quality of life, anxiety and depressive symptoms, and a negative profile of mood state as compared with wheelchair users practicing sports. However, we did not find significant differences between the non-athlete and athlete groups. Despite this, it is important to briefly describe the characteristics of our sample. We observed that athlete group had a statistically significant lower body mass than non-athlete group, which is a positive feature.

Concerning quality of life, the athlete group, in general, presented higher values in almost all factors evaluated. We can highlight the factors functional capacity and limitations by physical aspects. Regarding the anxiety-Trait and State levels, by the general mean of the non-athlete and athlete groups, we can observe that both groups present medium levels. With regard to depressive symptoms, by the general mean of the non-athlete and athlete groups, both groups present mild to moderate depression levels. Finally, with respect to the profile of mood state the athlete and non-athlete groups presented similar levels of vigor.

The participation in sports by people with disabilities can positively impact health status, levels of quality of life, physical fitness and psychological and emotional state (Bhambhani, 2002; De Lira et al., 2010; Blauwet and Willick, 2012; Côté-Leclerc et al., 2017; Lee and Uihlein, 2019). Kawanishi and Greguol (2013) performed a systematic review with the aim to study the influence of physical activity on the quality of life and functional independence of adult individuals with spinal cord injury (including wheelchair users). They found that this strategy has an important influence on social relationships, functional independence, psychological factors, and physical aspects, which can enhance quality of life and independence in the performance of daily activities.

In our study, although we found no significant differences between the groups that practiced and did not practice sports, this does not mean that the practice of sports has no beneficial effect on the quality of life and emotional well-being of wheelchair users. Richardson et al. (2017) demonstrated that sports participation in wheelchair tennis players may be a viable means to promote and enhance psychosocial well-being. These authors pointed out that skills learnt “on court” are transferrable to everyday life potentially improving independence and quality of life.

de Groot et al. (2013) showed that wheelchair users with a spinal cord injury (which, although not controlled, was the expressive part of our sample) generally had a relatively inactive lifestyle and they demonstrated that this situation was associated with a lower fitness level, poorer health, reduced social participation and a lower quality of life. However, an active lifestyle often requires a change in attitude or behavior and general practitioners, other primary healthcare providers and rehabilitation professionals can help in this respect.

Côté-Leclerc et al. (2017) showed that people with mobility limitations (9 women and 25 men with paraplegia, the majority of whom worked and played an individual adapted sport such as athletics, tennis or rugby at an international or national level, playing adapted sports and people without limitations had a similar quality of life. The authors demonstrated that participation in adapted sports was identified as having positive effects on self-esteem, self-efficacy, sense of belonging, participation in meaningful activities, society’s attitude toward people with mobility limitations, and physical well-being.

Although it was not possible to fully control the affections of the participants of the present study, a large part of the sample consisted of spinal cord injuries (i.e., tetraplegic and paraplegic). In this sense, Tweedy et al. (2017) showed that traumatic spinal cord injury may cause tetraplegia (i.e., motor and/or sensory nervous system impairment of the arms, trunk, and legs) or paraplegia (i.e., motor and/or sensory impairment of the trunk and/or legs only), which can lead to large negative effects on health, fitness and functioning and consequently to the sedentary behavior. In this sense, the benefits of practicing sport and physical exercise including reduced risk of depression and improvement of quality of life and functional independence which in a way we observed in our study despite the fact that it did not find statistical significance between groups of wheelchair users who practiced sports or not.

Finally, Muraki et al. (2000) examined whether the psychological benefits of sports activity differ between tetraplegics (i.e., poor mobility) and paraplegics with spinal cord injury. No significant difference was found for any psychological measurements between tetraplegics and paraplegics. However, they concluded that sports activity can improve the psychological status, irrespective of tetraplegics and paraplegics, and that the psychological benefits are emphasized by sports activity at high frequency.

### Study Limitations

Our study is not without limitations. Firstly, a factor that may have influenced the results is that the population of wheelchair used is very heterogeneous (both groups were composed of men and women) in terms of the causes and type and time of injury (which could not be controlled). Thus, studies with more homogeneous samples and group people by type of injury/affections are need. Also, the athlete wheelchairs were recruited from several places, such as therapeutic centers, rehabilitation clinics, universities and private companies. These are spaces of inclusion and social/health attention as well as the scenarios of the practice of sport. Nevertheless, all these limitations could consider non-fatal, since these are inherent difficulties in studies in this area.

## Conclusion

In conclusion, the wheelchair athletes and non-athletes presented similar quality of life, depressive and anxiety symptoms and profile of mood state.

## Data Availability

The datasets for this manuscript are not publicly available because the data are available on request from RLV. Requests to access the datasets should be directed to rodrigoluizvancini@gmail.com.

## Ethics Statement

All participants were informed about the aims, procedures, possible discomforts, risks, and benefits of the study, and signed the informed consent form. All the experimental procedures were submitted and approved by the Ethics Committee of the Federal University of Espírito Santo (CAAE: 70119817.3.0000.5542 and protocol number: 2.182.183) and are in accordance with the Declaration of Helsinki.

## Author Contributions

RLV, AG, HdPO, and CdL conceived and designed the study, collected the data, performed the statistical analysis, and drafted, edited, and revised the manuscript. WR-T, MA, KS, and MS collected the data, and drafted and revised the manuscript. RBV drafted, edited, and revised the manuscript. PN, TR, and BK edited and revised the manuscript.

## Conflict of Interest Statement

The authors declare that the research was conducted in the absence of any commercial or financial relationships that could be construed as a potential conflict of interest.
